# Supporting autistic adults’ episodic memory recall in interviews: The role of executive functions, theory of mind, and language abilities

**DOI:** 10.1177/13623613211030772

**Published:** 2021-07-09

**Authors:** Jade Eloise Norris, Katie Maras

**Affiliations:** 1 University of Bristol, UK; 2 University of Bath, UK

**Keywords:** autism spectrum disorders, autobiographical memory, cognition (attention, learning, memory), communication and language, episodic memory, executive functions, expressive language, interviews, task support hypothesis, theory of mind

## Abstract

Autistic people have difficulties recalling episodic memories, including retrieving fewer or less specific and detailed memories compared to typically developing people. However, the ability to effectively recall episodic memories is crucial in many real-world contexts, such as the criminal justice system, medical consultations, and employment interviews. Autistic people’s episodic memory difficulties are most apparent when open, unsupportive questions are used. The ‘Task Support Hypothesis’ posits that autistic people can recall as much information as typically developing people with more supportive questioning. Alongside problems retrieving episodic memories, autistic people also experience difficulties with executive functioning, theory of mind, and expressive language. The current study aimed to assess the impact of these abilities on recall in two previous studies by the authors that compared autistic and typically developing adults on recall specificity in police, healthcare, and employment interviews, and recall quality in employment interviews under unsupported and supported questioning. Under unsupported questioning only, autistic adults’ episodic autobiographical memory recall specificity was predicted by expressive language, whereas for the typically developing group, only theory of mind was a significant predictor. No other predictors were significant across the study. Implications for the task support hypothesis are discussed.

**Lay abstract:**

Autistic people have difficulties recalling *episodic memories* (memories of specific events) compared to typically developing people. However, being able to effectively recall such memories is important in many real-world situations, for example, in police interviews, during medical consultations, and in employment interviews. Autistic people’s episodic memory difficulties are most noticeable when they are responding to open, unsupportive questions. However, the ‘Task Support Hypothesis’ indicates that autistic people are able to recall as much information as typically developing people, as long as they are asked more supportive questions. Autistic people also experience difficulties with executive functioning (cognitive abilities which allow us to plan, hold information in mind, inhibit interruptions, etc.), theory of mind (the ability to understand others’ perspectives and intentions), and spoken language. The current study aimed to investigate the impact of these cognitive abilities on memory recall in two previous studies which compared autistic and typically developing adults on how *specific* their recall was in police, healthcare, and employment interviews, and the *quality* of responses during an employment interview when both unsupportive and supportive questioning was used. The results show that while typically developing people may rely on theory of mind abilities, autistic people may rely more on language abilities when performing in interviews, potentially to compensate for their episodic memory difficulties, and that this effect is most apparent during more unsupportive recall (e.g. when a brief, open question is asked) compared to when open questions are followed by prompts (e.g. ‘tell me about who as there’, ‘what happened?’, etc.).

Autism is a neurodevelopmental disorder characterised by difficulties in social communication and interaction, restricted interests and repetitive behaviours (American Psychiatric Association, 2013). In addition to these core clinically defining characteristics, an accumulating body of literature shows that autistic individuals also experience specific memory difficulties (see Desaunay et al., 2020, for a review). While autistic and typically developing (TD) individuals’ general autobiographical and personal semantic memories are broadly similar (i.e. personal facts or memories of extended periods of time; for example, a memory of a week’s holiday), autistic people have difficulties in recalling *episodic* memories (memories of specific events, Ben Shalom, 2003; Crane & Goddard, 2008; Goddard et al., 2007; Klein et al., 1999; McDonnell et al., 2017) and with episodic future thinking (e.g. Lind & Bowler, 2010; Lind et al., 2014; but see Crane, Lind, & Bowler, 2013). Specifically, compared to TD individuals, autistic adults retrieve fewer or less specific memories of particular instances they have experienced, with reduced detail and elaboration, and are slower to recall these memories (see Crane & Maras, 2018; Gaigg & Bowler, 2018).

Autobiographical memory (ABM) is proposed to serve important functions for the self, including in constructing and maintaining personal identity (Conway & Pleydell-Pearce, 2000), as well as a social function, such as in sharing memories to build and maintain relationships (e.g. Alea & Bluck, 2007), and a directive function, for problem solving and future planning (Bluck et al., 2005; Pillemer, 2003). ABM is also crucial to formal ‘high-stakes’ contexts, such as in recalling personally experienced incidents with sufficient accuracy and detail in interviews within the criminal justice system (e.g. of a witnessed theft), in consultations with medical professionals (e.g. describing how we fell over and hurt ourselves with sufficient specificity), and in employment interviews (e.g. conveying a favourable example of a time we worked in a team with sufficient relevant detail). Conveying specific, relevant episodic memories is an area of difficulty in autism. Indeed, when recalling narratives during an unsupportive task (i.e. one which provides few cues or explicit structure for recall, such as open-ended ‘tell me everything’ types of prompts), autistic people make more irrelevant and off-topic intrusions, and produce stories rated as less coherent which tend to depart from the main themes (e.g. Diehl et al., 2006; Losh & Gordon, 2014). Difficulties with appreciating the listener’s perspective, in terms of what they already know and what they need to know from the participant’s recall, have been suggested to impede the production of an appropriately detailed, coherent, and relevant account (Colle et al., 2008). Therefore, without careful consideration of questioning techniques, autistic people may not be sufficiently supported to provide all the relevant information to support their witness testimonies (Maras, in Press), to convey an incident/series of events with sufficient detail to support a medical diagnosis (e.g. Pohl et al., 2020), or to ‘sell’ themselves to an appropriate level in employment interviews (Maras et al., 2021).

Autistic people’s episodic memory difficulties are thought to be related to retrieval mechanisms, rather than problems with encoding, and are most prevalent when unsupportive questioning structures are used (e.g. in the criminal justice system, open, unsupportive questions such as ‘tell me everything that happened’ are ubiquitous; see Desaunay et al., 2020). Accordingly, the ‘Task Support Hypothesis’ posits that, with more supportive questioning (such as in cued recall or recognition tasks), autistic people can recall as much information as non-autistic people (Bowler et al., 1997, 2004). The explicit use of support (such as using specific questions and detailed instructions) has also recently been shown to reduce inaccurate and improve accurate reporting (Almeida et al., 2019; Maras et al., 2013, 2020; Mattison et al., 2015, 2018), improve recall specificity (i.e. of specific events with rich contextual detail), and relevance (Norris et al., 2020), and improve the quality of autistic adults’ responses in employment interviews (Maras et al., 2021).

Autistic people also experience executive functioning difficulties (e.g. Demetriou et al., 2018; Hill, 2004), which further compound their memory recall due to differences in retrieval strategies (Crane et al., 2009; Crane & Goddard, 2008; Dalgleish et al., 2007; Desaunay et al., 2020; Goddard et al., 2014; McCrory et al., 2007; McDonnell et al., 2017). For example, while TD adults can recall context and related pieces of information about an experienced event in a relatively effort-free, automatic way (cf. relational memory; see Gaigg & Bowler, 2018), autistic people may need to employ more effortful, strategic processing, therefore placing greater demands on executive functions (Maister et al., 2013). Indeed, in their study with autistic and TD children aged 11–13 years, Maister et al. (2013) reported no differences on executive functioning abilities between groups, but found that relational memory processing and episodic retrieval were related to visuospatial working memory (WM) and set-shifting ability for the autistic (but not the TD) group.

Although limited evidence currently exists in autistic populations, a range of executive functions have also been implicated in episodic memory retrieval in other populations, which may be pertinent to autism. For example, in older adults, recall specificity appears to be mediated by inhibition and updating (Piolino et al., 2010). Inhibitory control may be crucial to recall specificity by allowing the individual to inhibit irrelevant memories and details, and is also implicated in episodic memory retrieval failures in people with depression (Dalgleish et al., 2007; Raes et al., 2010). WM is proposed to facilitate the reconstruction of past events, or the simulation of future events (Lind & Bowler, 2010). Indeed, Crane et al. (2013) found that WM was associated with ABM recall specificity in autism, and proposed that this may be due to the role of WM in memory feature binding, another area of difficulty in autism (e.g. Bowler et al., 2011, 2014).

There is evidence that these executive functions may be diminished in autism, which has important implications for episodic memory recall and how this might be best supported. For example, several studies have reported that inhibitory control (Brady et al., 2017; Robinson et al., 2009; and see Geurts et al., 2014) and WM (Crane, Goddard, & Pring, 2013; Pennington & Ozonoff, 1996; Wang et al., 2017) are diminished in autistic compared to TD groups. However, other findings are mixed, with some studies finding similar performance between groups on inhibitory control (e.g. Geurts et al., 2004; Kana et al., 2007; Kleinhans et al., 2005; Lipszyc & Schachar, 2010; Pennington & Ozonoff, 1996) and WM (e.g. Geurts et al., 2004). Nevertheless, the presence or absence of group differences on tests of executive functioning do not necessarily preclude differential *use* of executive functions between groups during memory recall (see Maister et al., 2013), nor the need for support during recall to reduce executive processing demands. However, research to date has not systematically examined the association between these executive functions and episodic memory recall in autistic adults.

Alongside executive functions, social cognition difficulties may further compound autistic individuals’ episodic memory difficulties (e.g. Adler et al., 2010; Kristen et al., 2014), particularly with open, unsupportive questioning. Theory of mind (ToM) has been implicated in ABM retrieval in clinical samples such as those with schizophrenia (Corcoran & Frith, 2003); specifically, such individuals show a reduced propensity to selectively retrieve ABMs relevant to a conversation in order to relate to the other person’s experiences. Furthermore, metacognition, specifically metamemory, has been implicated in the link between ToM and broader memory abilities in autism, suggesting difficulties not only with theory of others’ minds, but with theory of own mind compounding recall difficulties (Grainger et al., 2014). Indeed, Kristen et al. (2014) found that autistic adults’ theory of *own* mind, but not ToM for *others’* minds, was related to episodic ABM recall. (Crane, Goddard, & Pring, 2013) also found a significant relationship between ToM and ABM recall in autistic adults, but the correlation became non-significant once IQ was controlled. Finally, Adler et al. (2010) found autistic participants’ ABM recall to be related to Reading the Mind in the Eyes test scores (non-significant for TD participants) but the relationship with Strange Stories performance was non-significant (significant for TD participants), suggesting that this pattern of relationships between groups may be due to autistic adults utilising more visual ABM recall during ToM, compared to TD adults, who may only utilise ABM during more complex reasoning tasks (Adler et al., 2010).

During communication, ToM plays a crucial role by allowing the interviewee to accurately gauge the task requirements from the interviewer. It has been suggested that autistic people’s poorer performance on open, unsupportive tasks may be due to ToM difficulties (e.g. Kenworthy et al., 2008; White, 2013; White et al., 2009). Indeed, Kenworthy et al. (2008) suggest that autistic adults’ difficulty in processing socially mediated task instructions results in experimenter-administered tests underestimating their executive abilities (particularly for more complex tasks), with performance often improved on computerised tasks (Kenworthy et al., 2008; Ozonoff, 1995). Relatedly, reduced narrative abilities, crucial to the verbal recall of ABMs, may also be linked to difficulties with ToM (Capps et al., 2000; Diehl et al., 2006; Losh & Gordon, 2014). Indeed, language abilities have been found to be related to episodic memory recall in autistic children (e.g. Boucher, 1981; Goddard et al., 2014), but the evidence in adults is more limited.

In sum, to recall high quality, specific, and relevant responses in a dynamic interview environment, it is necessary to monitor one’s own cognitions, plan an appropriate response, hold in mind various reporting options, inhibit the reporting of irrelevant or inaccurate details, and select an optimal level of detail, while considering one’s own and the interviewer’s perspective, all of which can be difficult for an autistic person. Thus, social demands and ToM difficulties may hinder autistic individuals’ performance in a range of formal social interaction contexts, from recalling eyewitnessed incidents under standard face-to-face questioning in the criminal justice system (Hsu & Teoh, 2017; Maras, in Press), and gauging appropriate information to report in healthcare consultations (Raymaker et al., 2017), to recalling specific personal information in employment interviews, where the desired response is often unclear (Maras et al., 2021).

Although researchers have begun to develop support for the difficulties faced by autistic people in recalling episodic memories across various applied contexts (Maras, in Press; Maras et al., 2020; Norris et al., 2020), the role of executive functions, social cognition, and language abilities requires direct exploration in order to further develop appropriate support. The current study aimed to assess the impact of these abilities on recall in two previous studies that compared autistic and TD adults on recall *specificity* in police, healthcare, and job interviews, and recall *quality* in employment interviews (Maras et al., 2021; Norris et al., 2020). Due to their proposed functions for episodic memory recall, we examined the impact of inhibition (Dalgleish et al., 2007), WM (e.g. Crane, Goddard, & Pring, 2013), ToM (Adler et al., 2010; Kristen et al., 2014), and expressive language (e.g. Capps et al., 2000) on recall performance. It was predicted that EFs, ToM, and language abilities would be related to recall when unsupportive questioning was used, compared to supportive questioning, and that this relationship would be more pronounced for the autistic compared to the non-autistic participants.

## Method

### Overview of original studies

Data were analysed from two previous studies by the authors testing adaptations to questions in order to support autistic adults’ episodic memory recall in different contexts, as outlined below (see Supplementary Materials A for further details of the measures in each study).

#### Recall specificity in police, healthcare, and employment interviews

Norris et al. (2020) tested the effectiveness of three levels of questioning support on the specificity and relevance of interviewees’ episodic ABM recall. In total, 30 autistic and 30 TD participants were asked a series of questions about personally experienced events that could be relevant to interviews in police (e.g. a time you have been to the bank), employment (e.g. a time you have worked as part of a team), and healthcare contexts (e.g. a time you have felt sad), with participants instructed to recall a specific instances in as much detail as possible. Levels of questioning support differed (within participants): in open (i.e. unsupportive) questions, participants were asked to recall an instance (‘tell me about . . .’) with no further prompting, while in a ‘visual-verbal prompting’ (V-VP) technique (supportive), initial open questions were immediately followed by specific prompts (e.g. ‘tell me about a time you went to the bank . . . Tell me when it happened, the setting, the people who were there, the actions that occurred, and any objects that were there?’ See Norris et al., 2020, for full details). Responses were coded for specificity on a 5-point scale (Piolino et al., 2002); for example, a score of 4 was given for the recall of a specific event (isolated, situated in time and space) with rich detail (e.g. actions, thoughts, perceptions, and images), whereas answers with no memory recalled scored 0 (see Norris et al., 2020). In the present study, we were interested in the relationships between executive functions, ToM, expressive language, and recall specificity in response to unsupportive open questions versus supportive V-VP questions, as autistic people provided less specific answers overall compared to TD participants, but V-VP task support was beneficial in improving specificity across all participants (Norris et al., 2020).

#### Recall quality in employment interviews

Maras et al. (2021) examined the efficacy of adapted employment interview questions for improving the quality of candidates’ recall. A total of 25 autistic and 25 TD participants underwent mock employment interviews, in two phases approximately 6 months apart. In Phase 1, participants were asked standard (unadapted, that is, unsupportive) employment interview questions (e.g. ‘Do you work well as part of a team?’). Employment professionals then rated the quality of participants’ responses from the interview transcripts, blind to group, and the questions were adapted to be more supportive based on professionals’ and participants’ feedback (predominantly by making questions more explicit in terms of the information required from interviewees, with prompts to help them structure their answers). 21 autistic and 21 TD participants returned for the Phase 2 interview with a counterbalanced set of adapted (i.e. supportive) questions (e.g. ‘I’m going to ask you to give me an example of a time you’ve worked in a team. What was your role in the team? How did you work with the other team members to solve problems?’), with each sub-question asked one at a time. Autistic participants’ responses were rated more poorly than TD participants with unsupportive questioning, but there was no group difference when supportive questions were used (Maras et al., 2021).

### Participants

In both studies, participants were recruited mainly from the South West of England and surrounding areas, including via previous research participation, autism-related and local community Facebook groups, social and support groups, and local community recruitment (including posters, magazine articles, and social media posts). All autistic participants had received a formal clinical diagnosis of an autism spectrum disorder according to the *Diagnostic and Statistical Manual of Mental Disorders* (4th ed.; DSM-IV; American Psychiatric Association, 2000) or DSM-5 criteria (American Psychiatric Association, 2013), and confirmed this with a copy of their clinical diagnostic report. Those who had received a diagnosis but were unable to access their report received the Autism Diagnostic Observation Schedule, Second Edition (ADOS-2; Lord et al., 2012) to confirm the diagnosis. All TD participants scored below the recommended minimum cut-off of 32 on the Autism Spectrum Quotient (AQ-50 with 80% specificity; Baron-Cohen et al., 2001). Participants provided their written informed consent to take part and were fully debriefed after each study. Ethical approval was obtained from the Psychology Research Ethics Committee at the University of Bath. Participant demographic information and scores for the dependent variables for each study can be found in the Supplementary Materials B.

### Materials

#### Inhibition

The Delis–Kaplan Executive Function System (D-KEFS; Delis et al., 2001) Colour Stroop task consists of a speed-reading phase (black ink colour-words), colour naming phase (naming the colour of squares), inhibition phase (naming the ink colour of incongruent colour words, such as ‘red’ printed in green), and a switching phase (switching between naming the ink colour or reading the incongruent-coloured word). The normed contrast score of the *inhibition phase* minus the *colour naming phase* was used to index inhibition, minimising the impact of processing speed (Coolin et al., 2014).

#### Working memory

The Corsi Block-Tapping Task (Corsi, 1973) is a spatial WM span task (computerised version via Inquisit; www.millisecond.com) consisting of a forward and backward span (Kessels et al., 2000, 2008). In up to eight trials of spans increasing by +1, participants were shown a visual array of nine blocks on a screen which ‘lit up’ in a fixed sequence. Participants were instructed to use the mouse to click the blocks in the same order as the lighting-up sequence (forward span) or in reverse order (backward span). The sequence length started at two, increasing by one up to a maximum of eight. The task terminated when participants gave incorrect responses to two trials of the same span length. Each participant’s longest backward span was used as an index of WM (Kessels et al., 2000, 2008). Online measures are reported to have good convergent validity with standard (i.e. non-online) Corsi block tasks (e.g. Siddi et al., 2020), and with split-half reliability reported to be moderate (0.78; de Paula et al., 2016).

#### Theory of mind

The Adult Theory of Mind (A-ToM) test (Brewer et al., 2017) was used to measure ToM. Following Brewer et al. (2017), participants watched six videos of social situations (e.g. involving *faux pas*, sarcasm, white lies, etc.) and six physical videos (situations which did not require consideration of mental states; for example, comparing interest rates offered by a bank and car finance) which played in a randomised order via Qualtrics. One question about what they had seen in the video was displayed on-screen immediately after the video ended, and participants were asked to type their response within 60 s. Participants’ answers were rated by two independent raters on a 0–2 scale: 0 (incorrect), 1 (partially correct), or 2 (correct) (see Brewer et al., 2017, for scoring criteria). Each rater pair met to discuss discrepancies in coding and agreed all final scores. The test–retest reliability is reported at *r* = 0.82 for the physical scale, and *r* = 0.64 for the social scale (Brewer et al., 2017).

#### Expressive language

The Expression, Reception and Recall of Narrative Instrument (ERRNI; Bishop, 2004) was used to measure expressive language. For the ERRNI task, participants were provided with a wordless picture book and asked to silently view all the images until they had the story in their mind. They were then asked to narrate the story aloud, following the pictures. Then, after 15–30 min, the participants were again asked to tell the story out loud *without* looking at the picture book. The current study focuses on the syntactic complexity of expressive language, indicated by mean length of utterances scores (MLU; the mean number of words in each utterance) from the recall trial.

### Procedure

The current study included data from 57 participants who were administered tests of executive functioning, ToM, and language during the studies, conducted over a 2-year period. In total, 33 of the participants (18 autistic, 15 TD) took part in both studies, and 24 in the ABM study only (10 autistic, 14 TD). The tests were administered after the episodic memory task in each study, and participants were able to take breaks when required. During the study of *Recall specificity in police, healthcare, and job interviews*, participants were administered the ERRNI, inhibition, WM, and ToM tasks. For the *Recall quality in employment interviews* study, the inhibition and WM tests were administered to any participants who had not previously completed them.

### Community involvement

Community involvement was not included in the current study.

## Results

### Analysis plan

In order to understand the contribution of executive functions, ToM, and expressive language abilities to episodic memory recall, multiple linear regression analyses were conducted for each group (autistic vs TD) in both studies (ABM specificity and employment recall quality). Due to their role in memory recall, inhibition, WM backward span, A-ToM social score, and ERRNI MLU were included as predictors in each of the analyses, which were conducted separately for the TD and autistic groups.

### Recall specificity in police, healthcare, and employment interviews

Inspection of normality plots indicated that the specificity data (both unsupported and supported) for TDs was positively skewed, deviating from a normal distribution. The data were therefore log transformed. In addition, a low scoring outlier was removed for the Stroop inhibition data for the TD group (score of 3; 3.50 SDs below the group mean). For the autistic group, one low-scoring outlier was removed from the supported specificity data (score of 1.67; 3.65 SDs below the group mean). See Supplementary Materials C and D for descriptive statistics and group difference statistics on measures of executive functioning, ToM, and expressive language abilities in each study.

Four multiple linear regressions were conducted, predicting specificity with and without support separately for the autistic (*n* = 28) and TD (*n* = 29) groups (see Table 1).

**Table 1.** Regression analyses for predictors of autobiographical memory recall specificity under unsupported and supported questioning.

	*b*	*SE b*	*β*
TD unsupported			
Constant	1.69	1.58	
Inhibition	−0.05	0.05	−0.18
WM	0.03	0.08	0.07
A-ToM	1.49	0.42	0.63*
ERRNI MLU	−0.00	0.01	−0.11
TD supported			
Constant	2.11	1.24	
Inhibition	0.04	0.04	0.17
WM	0.05	0.06	0.15
A-ToM	0.63	0.33	0.37
ERRNI MLU	−0.00	0.01	−0.07
Autistic unsupported			
Constant	1.52	0.88	
Inhibition	−0.08	0.05	−0.26
WM	0.07	0.09	0.14
A-ToM	0.24	0.24	0.17
ERRNI MLU	0.01	0.01	0.54*
Autistic supported			
Constant	2.51	0.61	
Inhibition	−0.01	0.04	−0.05
WM	0.07	0.06	0.25
A-ToM	0.08	0.17	0.10
ERRNI MLU	0.01	0.00	0.31

SE: standard error; TD: typically developing; WM: working memory; A-ToM: adult theory of mind; ERRNI: Expression, Reception and Recall of Narrative Instrument; MLU: mean length of utterances.

* *p* < 0.05.

#### TD group

*Unsupported*: The model was significant (*p* = 0.025), accounting for 36% of the variance in specificity. ToM (*p* = 0.002) predicted specificity. All other predictors were non-significant (*p*s > 0.263).
*Supported*: The overall model was non-significant (*p* = 0.136).

#### Autistic group

*Unsupported*: The model was significant (*p* = 0.015), accounting for 40% of the variance in specificity. expressive language predicted specificity (*p* = 0.003). All other predictors were non-significant (*p*s > 0.148).
*Supported*: The model was non-significant (*p* = 0.270).

In summary, for TD adults, ToM predicted specificity when questions were unsupportive, but was no longer a significant predictor with support. For the autistic group, expressive language predicted specificity in response to unsupportive questioning, but was no longer a significant predictor with support.

### Recall quality in employment interviews

Outlying data for one participant in the autistic group were removed from the analyses (supported answer ratings 3.25 SDs below the mean). Four multiple linear regressions were conducted predicting employers’ ratings of the quality of participants’ responses separately for unsupported and supported questioning for the autistic (*n* = 18) and TD (*n* = 15) groups, with all predictors as above.

*All models were non-significant*: unsupported TD (*p* = 0.584), and autistic (*p* = 0.497), and supported TD (*p* = 0.524), and autistic (*p* = 0.876; see Table 2).

**Table 2.** Regression analyses for predictors of recall quality ratings in unsupported and supported mock employment interviews.

	*b*	*SE b*	*β*
TD unsupported			
Constant	6.77	3.41	
Inhibition	−0.13	0.09	−0.53
WM	0.05	0.24	0.06
A-ToM	0.37	0.89	0.14
ERRNI MLU	−0.02	0.02	−0.44
TD supported			
Constant	5.17	2.44	
Inhibition	−0.06	0.06	−0.35
WM	0.21	0.17	0.36
A-ToM	0.05	0.64	0.03
ERRNI MLU	−0.02	0.01	−0.44
Autistic unsupported			
Constant	3.27	1.20	
Inhibition	−0.04	0.05	−0.22
WM	−0.10	0.12	0.22
A-ToM	0.24	0.28	0.21
ERRNI MLU	0.01	0.01	0.33
Autistic supported			
Constant	3.89	1.33	
Inhibition	−0.01	0.06	−0.06
WM	−0.07	0.13	−0.15
A-ToM	0.27	0.31	0.24
ERRNI MLU	0.00	0.01	0.02

SE: standard error; TD: typically developing; WM: working memory; ERRNI: Expression, Reception and Recall of Narrative Instrument; MLU: mean length of utterances.

## Discussion

The current study investigated the extent to which executive functioning, ToM, and expressive language abilities predict specificity and quality of autistic and TD adults’ episodic memory recall under unsupportive and supportive questioning. Specifically, the contributions of WM, inhibition, ToM, and expressive language to recall performance were assessed. In line with the mixed findings in previous research regarding group differences in executive functions and language abilities, in the current study, the autistic group did not perform more poorly than the TD group in terms of WM, inhibition, or expressive language (e.g. Geurts et al., 2004; Kana et al., 2007; Kleinhans et al., 2005; Lipszyc & Schachar, 2010; Pennington & Ozonoff, 1996). As expected, however, autistic adults scored significantly below TD adults on the A-ToM social scale (Brewer et al., 2017). In terms of predictors of memory performance, ToM predicted TD participants’ specificity during ABM recall in response to unsupportive questioning, whereas expressive language significantly predicted the autistic group’s performance. When questions were supportive, executive functions, ToM, and expressive language did not significantly predict specificity for either group. Executive functioning, ToM, and language did not predict employment interview response *quality* for either group when questions were supportive or unsupportive. The findings are discussed in turn below.

In terms of recalling specific ABMs, the current findings suggest that while TD interviewees recruit ToM when generating episodic ABMs in response to unsupportive questions, autistic adults utilise expressive language abilities. Some degree of ToM is suggested to be required during specific ABM recall, as participants are recalling a personally relevant event, and need to for example, provide details about their own and others’ actions and thoughts (Kristen et al., 2014). Furthermore, ToM may play a broader role (for TD individuals at least) in gauging the expectations of the interviewer, which is likely to have been particularly appropriate in this study, which involved a face-to-face interview (e.g. Kenworthy et al., 2008; White, 2013; White et al., 2009).

In contrast, rather than relying on (diminished) ToM to formulate a response, autistic adults may rely more upon expressive language abilities during unsupportive questioning. This may be due to the highly verbal nature of the task (verbally recalling an ABM during an in-person interview; see Desaunay et al., 2020). Expressive language abilities have been found to be related to episodic memory recall in autistic children (e.g. Boucher, 1981; Goddard et al., 2014), but little research has investigated this in autistic adults. Although the current study found no group differences on this measure, the findings highlight differential recruitment of cognitive abilities between autistic and TD groups during unsupported recall (e.g. Maister et al., 2013). As a coherent narrative is crucial to recall performance (e.g. Diehl et al., 2006; Losh & Gordon, 2014), future research examining the role of narrative abilities in episodic memory recall with varying task demands and questioning support could prove fruitful.

The findings with regard to ToM are in contrast to those of (Crane, Goddard, & Pring, 2013), who found that ToM was related to ABM specificity for the autistic group only (and this relationship became non-significant when controlling for IQ). The relatively new ToM measure utilised in the current study is worth consideration in this regard (the A-ToM; Brewer et al., 2017), which detected poorer performance in the autistic group compared to TD participants in the current study, a difference that prior studies using other ToM tasks has often failed to detect (e.g. Bowler, 1992; Ozonoff et al., 1991, but see Livingston et al., 2019; Murray et al., 2017). Moreover, Adler et al. (2010) found different relationships between ToM and ABM recall for autistic and TD groups depending on task type (autistic participants’ ABM recall was related to Reading the Mind in the Eyes test scores, but recall was related to Strange Stories performance for TD participants). Future research may therefore benefit from investigating the potential role of task type in relationships between ToM and ABM specificity. When questions were supportive, performance was not significantly related to any of the measures for either group, indicating that when supportive questioning is used, ABM recall may not require significant recruitment of executive functions, ToM, or expressive language by autistic or TD adults. These findings support the beneficial effect of adapting questions to support memory recall (in line with the task support hypothesis; Bowler et al., 1997).

For the employment interviews study, answer quality during unsupported and supported questioning was not predicted by executive functioning, ToM, or language abilities for either group. In the reference study (Maras et al., 2021), autistic adults were rated more poorly than their TD counterparts in the unsupported condition, but this difference was ameliorated when supportive questions were used. The lack of significant predictors may not be a surprising finding: the employment interviews study could be considered to be more generally supportive than the ABM study, particularly for the supported condition, whereby questions were separated into shorter questions and prompts, and participants were given a paper copy of the questions to refer to during the interview, potentially scaffolding participants’ ability to monitor their responses (Lind & Bowler, 2010). Moreover, support was tailored to each question, providing bespoke guidance on ideal answer content and therefore an even greater degree of task support (Bowler et al., 1997). On the other hand, for the ABM study, although the questions in the supportive condition provided specific prompts (‘tell me about when it happened, the people who were there, the actions that occurred . . .’), these were general prompts used across all questions, with the task requiring the participant to verbally narrate a personal memory in detail. This may have resulted in reduced reliance on executive functions, ToM, and language abilities during the more supportive mock employment interview. In addition, although specific memories were emphasised as important to answers in the employment interview (i.e. relaying relevant instances of meeting a deadline, working as a team, etc.), employers’ ratings were based on more than memory specificity, as opposed to the ABM study. Overall, it could therefore be suggested that both studies analysed here may be generally more supportive than other previous studies of episodic recall in autism, even in the designated ‘unsupportive’ conditions (see Semino et al., 2018, for similar findings for object recognition and source memory). It is therefore possible that our paradigms were already sufficiently supportive, reducing the necessity to recruit executive functions during recall, including for example the overall limited use of open questions, the provision of printed question sets (in the employment interviews supportive condition) and clear recall parameters for participants (V-VP diagram in the ABM supportive condition). Future research should examine the contributions of executive functions, ToM, and language abilities on recall in less structured, free recall tasks (e.g. requiring witnesses to produce a detailed narrative account to a single ‘tell me everything that happened’ prompt), in order to determine whether executive functions are recruited as compensatory mechanisms in such instances.

Overall, the findings provide further evidence for the role of executive functions, ToM, and expressive language in episodic memory recall (Adler et al., 2010; Crane et al., 2009; Crane & Goddard, 2008; Dalgleish et al., 2007; Goddard et al., 2014; Kristen et al., 2014; McCrory et al., 2007; McDonnell et al., 2017), adding to previous findings in showing that reliance on additional cognitive abilities may be mediated by the degree of task support. Specifically, the current findings highlight the subtle differences between levels of questioning support provided in traditional compared to adapted, supportive questions in applied contexts. The findings have implications for the further development of support for autistic people in recalling past experiences across various real-life contexts such as interviews and consultations. Recent research is beginning to uncover methods which may be utilised to improve specific episodic memory recall in autistic adults, possibly due in part to the reduction of executive load and the reduced role of language abilities with more supportive questioning. For example, following-up open questions with specific prompts (e.g. ‘tell me about a time you went to the bank?’ followed by, for example, ‘(tell me) when it happened, the setting, the people who were there . . .’), which are also visible to the participant during recall, improve the specificity and relevance of autistic and TD adults’ recall (Norris et al., 2020). Furthermore, providing question-specific adaptations during employment interviews reduces differences in terms of answer quality as rated by employers (Maras et al., 2021). Future research may therefore aim to target support to relevant executive functions and language abilities based upon the type of recall, the degree of unstructured questioning present in the task, and its real-world context.

The current study has several important limitations that need to be borne in mind when interpreting the findings. Due to the two-part nature of the employment interviews study, this aspect of the current study contained a smaller than desired sample size, restricting our ability to draw firm conclusions regarding the effects of ToM, executive functions, and expressive language abilities on employment interview performance, and (although to a lesser extent), the recall specificity in police, healthcare, and employment interviews study. Furthermore, the current study did not have the scope to investigate gender differences in memory recall. Although sex differences are found in some studies of ABM (e.g. Grysman & Hudson, 2013; Herlitz & Rehnman, 2008; Schulkind et al., 2012), findings regarding sex differences on dependent variables similar to those of interest here (e.g. specificity) are mixed (Baron & Bluck, 2009; Bluck et al., 2005; Q. Wang, 2004). Future research should aim to directly investigate the additional impact of sex differences on memory recall differences between autistic and non-autistic adults, and the relationship with ToM, executive functions, and expressive language. Finally, it has been proposed that using standardised scores may reduce the predictive power of the D-KEFS executive functioning tasks (inhibition), as participants with varied raw scores are often classified under the same standardised score (Henry et al., 2017). However, as our sample represented a wide adult age range (18–60 years), standard scores were necessary to enable comparisons between groups on this measure. Future research should seek to understand the specific executive functions underlying episodic recall, particularly for individuals with poorer executive functions, who may be at an even greater disadvantage in important recall contexts such as interviews and consultations.

## Supplemental Material

sj-docx-1-aut-10.1177_13623613211030772 – Supplemental material for Supporting autistic adults’ episodic memory recall in interviews: The role of executive functions, theory of mind, and language abilities Click here for additional data file. Supplemental material, sj-docx-1-aut-10.1177_13623613211030772 for Supporting autistic adults’ episodic memory recall in interviews: The role of executive functions, theory of mind, and language abilities by Jade Eloise Norris and Katie Maras in Autism
