# Supplementary material for: Supporting autistic adults’ episodic memory recall in interviews: The role of executive functions, theory of mind, and language abilities
Source: Autism. 2021 Jul 9;26(2):513–24. doi: 10.1177/13623613211030772 (PMC8814938; doi:10.1177/13623613211030772)
Supplement: sj-docx-1-aut-10.1177_13623613211030772 – Supplemental material for Supporting autistic adults’ episodic memory recall in interviews: The role of executive functions, theory of mind, and language abilities [file sj-docx-1-aut-10.1177_13623613211030772.docx]

## Supplementary Materials

### Supplementary Materials A. Summary of interview type, unsupported and supported questioning examples, and the dependent variables for each study

| **Study reference** | Norris et al. (2020) | Maras, Norris, Nicholson, et al. (2020) |
| --- | --- | --- |
| **Interview type^[[1]](#footnote-1)^** | Autobiographical memory interview, recalling personally-experienced episodic memories relevant to police interviews (events where crimes could happen; e.g., a memory of going to a party), healthcare consultations (memories related to physical and mental health difficulties), and employment interviews (social and non-social work-related memories, e.g., working as a team, meeting a deadline) | Mock employment interviews, whereby participants were asked commonly-used work-related questions such as ‘What are some of your strengths?’, and ‘Tell me about a time you had to work with someone who was difficult to get along with – how did you handle it?’ across two Phases (unsupported and supported, see below) |
| **Unsupported questioning example** | “Tell me about a time you went to a party?” | “What are some of your strengths?’ |
| **Supported questioning example** | “Tell me about a time you went to a party - Tell me about **when** it happened, the **setting**, the **people** who were there, the **actions** that occurred, and any **objects** that were there?”  (Participants had a diagram with the bolded words in front of them) | “I’m going to ask about your strengths: What do you consider to be your main strengths (things that you are good at)?” [participant answers] “How have you used these strengths at work?” [participant answers]  (Participants had a copy of the questions in front of them) |
| **Scoring and dependent variable** | Specificity  Scored on a scale of 0 (absence of a memory) to 4 (memory is highly specific, clearly situated in time and space) | Quality (employer rated)  Scored on a scale of 1 (very poor; a single yes/no answer with little to no elaboration/irrelevant/off-topic response) to 5 (excellent; fully satisfied all aspects of the question, relevant to employment) |

### Supplementary Materials B. Mean age, gender, Wechsler Abbreviated Scale of Intelligence (WASI-II), and AQ scores by group for each study (standard deviations in parentheses)^[[2]](#footnote-2)^

|  | *Autobiographical memory specificity and relevance* | | *Employment interview recall quality* | |
| --- | --- | --- | --- | --- |
|  | **TD (N = 29)** | **Autistic (N = 28)** | **Autistic (N = 18)** | **TD (N = 15)** |
| Gender | 8 males, 21 females | 15 males, 11 females, 2 genderfluid/no preference | 11 males, 7 females | 4 males, 11 females |
| Age (years) | 35.10 (13.47) range = 18-59 | 33.82 (12.22) range = 18-58 | 35.22 (12.29) range = 18-53 | 34.60 (11.26) range = 18-50 |
| Verbal IQ | 108.83 (8.67) range = 94-142 | 107.14 (10.58) range = 85-128 | 106.61 (9.10) range = 85-121 | 108.67 (6.75) range = 94-118 |
| Performance IQ | 113.76 (11.12) range = 92-136 | 108.54 (12.55) range = 82-131 | 107.67 (13.91) range = 82-131 | 112.13 (11.90) range = 92-136 |
| Full-scale IQ | 112.69 (7.46) range = 95-126 | 108.82 (11.27) range = 89-129 | 108.06 (11.07) range = 91-129 | 111.73 (8.18) range = 95-123 |
| AQ-50 | 13.83 (8.67) range = 2-30 | 36.22 (7.51) range = 21-48^[[3]](#footnote-3)^ | 36.24 (7.43)  range = 20-46 | 10.53 (7.62) range = 2-24 |
| Specificity (unsupported)^[[4]](#footnote-4)^ | 0.52 (0.07), range = 0.34-0.60, n = 29 | 3.08 (0.60); range = 1.83-4.00, n = 28 | - | - |
|  |  |  |  |  |
| Specificity (supported) | 0.56 (0.05), range = 0.45-0.60, n = 29 | 3.51 (0.36); range = 2.83-4.00, n = 28 | - | - |
| Answer quality (unsupported) | - | - | 3.33 (0.45); range = 2.64-4.32; n = 18 | 3.89 (0.56); range = 2.75-4.75; n = 15 |
|  |  |  |  |  |
| Answer quality (supported) | - | - | 3.80 (0.46); range = 3.13-4.71; n = 18 | 4.16 (0.41); range = 3.32-4.71; n = 15 |

### Supplementary Materials C. Group differences for executive functioning, ToM, and language measures for participants in the Recall specificity in police, healthcare, and employment interviews study

|  | TD (N = 29) | Autistic (N = 28) | t tests |
| --- | --- | --- | --- |
| Inhibition: Stroop inhibition | M ­= 11.66 (SD = 1.82); Range = 7–15 | M ­= 11.39 (SD = 1.95); Range = 6-14 | t(55) = 0.53, p = .601, d = 0.14 |
| Spatial working memory: Backward span | M ­= 5.97 (SD = 1.09); Range = 3-7 | M ­= 5.64 (SD = 1.19); Range = 3-7 | t(55) = 1.07, p = .290, d = 0.29 |
| Theory of Mind:  A-ToM Social | M ­= 1.73 (SD = 0.21); Range = 1.33-2.00 | M ­= 1.47 (SD = 0.43); Range = 0.33-2.00 | t(38.76) = 2.85, p = .007, d = 0.77 |
| Language: ERRNI Mean Length of Utterances | M ­= 114.14 (SD = 12.58); Range = 87–135 | M ­= 112.54 (SD = 21.17); Range = 65–135 | t(43.66) = 0.35, p = .731, d = 0.09 |

###

### Supplementary Materials D. Group differences for executive functioning, ToM, and language measures for participants in the Recall quality in employment interviews study

|  | TD (n = 15) | Autistic (n = 18) | t tests |
| --- | --- | --- | --- |
| Inhibition: Stroop inhibition | M ­= 11.53 (SD = 2.32) Range = 7–15 | M ­= 11.67 (SD = 2.33); Range = 6-14 | t(31) = -0.16, p = .871, d = 0.06 |
| Spatial working memory: Backward span | M ­= 6.27 (SD = 0.70); Range = 5-7 | M ­= 5.83 (SD = 0.99); Range = 3-7 | t(31) = 1.43, p = .164, d = 0.51 |
| Theory of Mind:  A-ToM Social | M ­= 1.71 (SD = 0.22); Range = 1.33-2.00 | M ­= 1.48 (SD = 0.40); Range = 0.50-2.00 | t(27.20) = 2.06, p = .050, d = 0.71 |
| Language: ERRNI Mean Length of Utterances | M ­= 113.47 (SD = 11.91); Range = 94–135 | M ­= 115.89 (SD = 21.20); Range = 65–135 | t(27.52) = -0.41, p = .683, d = 0.14 |

1. See original papers for full methods [↑](#footnote-ref-1)
2. Groups were matched on age and IQ in both studies (all *p*s > .05) [↑](#footnote-ref-2)
3. AQ data for one autistic participant were not available. It is noted that 9 autistic participants in the *Autobiographical memory specificity and relevance* study, and 5 autistic participants in the *Employment interview recall quality study* scored below the advised clinical cut-off score of 32 on the AQ. This is in line with research indicating that AQ scores did not significantly predict autism diagnosis (Ashwood et al., 2016) . Indeed, Ashwood et al. (2016) found that 64% of their sample scoring below the AQ cut-off received an autism diagnosis. [↑](#footnote-ref-3)
4. Specificity data are log transformed for the TD group [↑](#footnote-ref-4)
